# Antimicrobial, Time–Kill Kinetics, and Biofilm Inhibition Properties of *Diospyros lycioides* Chewing Stick Used in Namibia Against *Enterococcus faecalis*

**DOI:** 10.1155/jotm/7544856

**Published:** 2025-06-16

**Authors:** Albertina Mariina Ndinelao Shatri, Silas Kudakwashe Bere, Denise Bouman, Davis Ropafadzo Mumbengegwi

**Affiliations:** ^1^Department of Human Biological & Translational Medical Sciences, School of Medicine, Hage Geingob Campus, University of Namibia, Windhoek, Namibia; ^2^Department of Oral Surgery, School of Dentistry, Hage Geingob Campus, University of Namibia, Windhoek, Namibia; ^3^Multidisciplinary Research Services, Centre for Research Services, University of Namibia, Windhoek, Namibia

**Keywords:** antibacterial activity, antioxidants, biofilm inhibition, kill time

## Abstract

**Background:** Medicinal plants are used in Namibia for oral hygiene and to treat oral diseases. Validating the content and efficacy of medicinal chewsticks used in communities helps to provide proof of concept of medicinal plants used as a complementary/alternative medicine for oral diseases.

**Aim:** This study presents the first report on quantified phytoconstituents, antimicrobial, time–kill kinetics, and biofilm inhibition properties of *Diospyros lycioides* organic and aqueous extracts against *Enterococcus faecalis*.

**Methodology:** Dry plant materials were ground into powder and macerated in methanol and distilled water. Different phytoconstituents were quantified by Folin–Ciocalteu colorimetric method, ferric reducing antioxidant power assay, and DPPH free radical scavenging. An antibacterial assay was performed using the agar well diffusion method and a resazurin 96-well-based assay. Kill–time assay was done at various concentrations over 4 h. Biofilm inhibition was done using the crystal violet method.

**Results:** Higher total flavonoid, total phenol contents, and free radical scavenging abilities were reported in methanol twig extracts. Inhibition zones of 28 ± 0.82 mm, with MICs of 15.6 ± 0.00 μg/mL, are reported against *E. faecalis*. The bactericidal endpoint of *D. lycioides* organic extracts for *E. faecalis* was reached after 4 h of incubation at 8 × MIC (124.8 μg/mL). These were comparable to the positive control, gentamicin. The organic extracts showed ≥ 50% biofilm inhibition against root canal-infecting *E. faecalis* at concentrations between 7.8 and 500 μg/mL, indicating strong biofilm inhibition.

**Conclusion:** The study demonstrated that *D. lycioides* crude extracts have promising antibacterial properties and can eradicate *E. faecalis* biofilms in root canal treatments.

## 1. Background

Oral hygiene is one of the most neglected practices in many African countries; however, oral diseases are increasingly being recognized as a major public health problem in Africa, where about 400 million people suffered from oral disease in the African Region in 2017 [1]. Up to 60% of dental infection cases are reported annually, with healthcare costs exceeding $105 million globally.

*Enterococcus faecalis* (*E. faecalis*) is the most frequent species in root canals of endodontically treated teeth, with the prevalence reaching up to 90% of cases [2]. The goal of root canal treatment is to prevent the development of apical periodontitis, by removing infected and inflamed pulpal tissues while creating aseptic intra-articular conditions compatible with periarticular healing of any existing lesion. Failure of root canal treatment is mainly attributed to the eradication of bacteria and incomplete disinfection of the complex root canal system, which normally results in persistent apical periodontitis [3]. *E. faecalis* is a Gram-positive bacterium that commonly colonizes the root-filled teeth of patients with chronic apical periodontitis.

*E. faecalis* invades the dentinal tubules and adheres to the root canal wall, forming a biofilm on dentin. *E. faecalis* can deeply penetrate the dentinal tubules to a depth of 1500 μm, which makes it unreachable by several first-line intracanal medications such as sodium hypochlorite, clindamycin, calcium hydroxide, ciprofloxacin, metronidazole, and sealers [4]. Moreover, resistance has been reported to antibiotics such as quinupristin, dalfopristin, penicillin, and vancomycin. This resistance is due to the formation of bacteria biofilm that allows it to survive even in a nutrient-depleted environment such as root-filled teeth [5]. This results in chronic infections and higher antimicrobial resistance caused by poor diffusion of the double antibiotic pastes. While gentamicin is currently one of the antibiotics used for *E. faecalis* infections, clinical studies have reported a 50% resistance in *E. faecalis* isolated from 155 patients [6]. This is due to the lower uptake of gentamicin within the root canal and its inability to eliminate *E. faecalis* biofilm. This then requires multiple dosages of the antibiotic, which in return contributes to severe side effects such as toxicity and increases the chances of resistance [7].

Medicinal plants have a long history of use in African and Asian countries [8, 9]. Approximately 8000 naturally occurring compounds known as phenolics are linked to the therapeutic benefits of medicinal plants [10]. These hydroxyl group compounds are the custodians of different biological activities such as antioxidants and antibacterial properties referred to as the index of phytomedicine. They have also been involved in diverse functions, including protein synthesis, enzyme activity, structural components, and allelopathy [11]. *D. lycioides* has a long ethnomedicinal history in Namibia to treat oral infections. Local people use the twigs and roots as toothbrushes, and the leaves are chewed for general oral hygiene [12]. *D. lycioides* contain various phytocompounds such as alkaloids, terpenoids, coumarins, and phenolic groups of compounds; however, there is limited information regarding the quantities of such phytoconstituents, and antioxidant properties [12]. This is important as it helps to provide information regarding the compounds that may be responsible for the efficacy of *Diospyros lycioides* (*D. lycioides*). These compounds are documented to use various mechanisms such as inhibiting microbial enzymes and disrupting bacterial cell walls, weakening bacterial defenses (flavonoids and tannins), interfering with bacterial membrane integrity, leading to cell death (terpenoids), generating oxidative stress, and damaging microbial DNA and proteins (phenolic compounds). Although the preliminary screening of phytochemical compounds has been done through our other studies [12], there are no data on the total quantification and antioxidant properties of *D. lycioides*. Other studies have shown that plants with higher quantities of phytochemical compounds have potent antimicrobial properties since such compounds express significant interactions with pathogens through multiple mechanisms offering diverse mechanisms of action [13]. Hence, *D. lycioides* could serve as a source of phytoconstituents with therapeutic and free radical scavenging potential. To the best of our knowledge, the phytochemical composition of *D. lycioides* has not been quantified, and the antioxidant, antibacterial, and biofilm eradication properties of *D. lycioides* are also not documented. This study presents the first findings aimed at filling this identified knowledge gap.

## 2. Methods

### 2.1. Plant Collection and Processing

*D. lycioides* used in this study were identified through an ethnobotanical approach and scientifically authenticated by the Namibian Botanical Institute (NBRI) voucher specimen number TONI2. The information on its use and preparation in Namibia was gleaned from knowledge holders. The plant is being investigated based on its ethnomedicinal uses to treat oral diseases and as a daily toothpick used to brush teeth by community members. This plant is not an endangered species, and it was collected in an open community field after permission from the National Commission on Research Science and Technology (NCRST). Different plant parts (roots, leaves, and twigs) were collected and used in this study. The ethical approval to conduct this study was obtained from the University's School of Dentistry ethical committee in September 2023 (Certificate Number: SOD 0059). Roots, twigs, and leaf plant materials of *D. lycioides* were rinsed with tap water followed by distilled water to remove the dirt on the surface. The plant parts were cut into small pieces and air-dried for 30 days in the shade. The dried roots, twigs, and leaf plant materials of *D. lycioides* were ground into a fine powder using an industrial blender and stored in closed sterile containers until extraction [9].

### 2.2. Preparation of Aqueous Crude Extracts

Briefly, 20 g of powdered roots, twigs, and leaves of *D. lycioides* were macerated in 100 mL of distilled water for 24 h. The macerations were kept on a shaker at 60°C for 1 h. The extracts were filtered using Whatman No. 1 filter papers with a diameter of 110 mm to collect the filtrates. The aqueous filtrates are further dried into powder by rotary evaporation and freeze-drying for 5 days [14, 15]. The dry crude aqueous extracts were weighed and stored at 4°C until further use. The % yield was calculated using the formula: yield (%) = (weights of solvent-free aqueous crude extract (g) × dried plant material weight (g)) × 100. The above experiment was repeated several times to increase the extract yield for more biological assays.

### 2.3. Preparation of Organic Crude Extracts

Briefly, 20 g of powdered roots, twigs, and leaves of *D. lycioides* were macerated in 100 mL of 99% methanol for 24 h to prepare organic extracts. The macerations were kept on a shaker at 60°C for 1 h. The organic extracts were filtered using Whatman No. 1 filter paper with a diameter of 110 mm to collect the filtrations. The organic filtrates were further dried into powder by rotary evaporation and freeze-drying for 5 days [14, 15]. The dry organic crude extracts were weighed and stored at 4°C until further use. The % yield was calculated using the formula: yield (%) = (weights of solvent-free organic crude extract (g) × dried plant material weight (g)) × 100. The above experiment was repeated several times to increase the extract yield for more biological assays.

### 2.4. Estimation of Total Phenolic Content

The total phenolic content of *D. lycioides* extracts was determined using the Folin–Ciocalteu technique using gallic acid as a standard, and the results were represented in milligrams of gallic acid equivalents (mg GAE/g) of sample dried weight using the method by Akbar et al. [16]. Exactly 0.5 mL at a concentration of 1 mg/mL dried crude plant extract was added to 2 mL Folin–Ciocalteu reagent. The mixture was incubated for 5 min at room temperature and neutralized with 2 mL of 10% Na_2_CO_3_ before being further incubated for 30 min. The absorbance was read at 750 nm using a UV–visible spectrophotometer. Exactly 95% of methanol was used as a blank. Gallic acid was used as a positive control, and the experiment was repeated 3 times.

### 2.5. Estimation of Total Flavonoid Content

The total flavonoid contents of *D. lycioides* were determined by aluminum chloride colorimetric assay using the method by Akbar et al. [16]. Quercetin was used to develop a calibration curve, and the results were recorded in mg QE/g. Exactly 0.5 mL of the extract (1 mg/mL) was mixed with 95% methanol and 0.5 mL (NaNO_2_ 5%) solution. After 5 min of incubation, exactly 10% w/v, 0.1 mL of AlCl_3_.6H_2_O, 0.5 mL of 1M NaOH, and 2 mL of deionized water were added and incubated at 25°C for 40 min. Absorbance was measured at 415 nm against a blank using a UV–visible spectrophotometer. The experiment was repeated three times.

### 2.6. Determination of Antioxidant Properties

The antioxidant activity of *D. lycioides* organic and aqueous extracts was determined using DPPH° scavenging and reducing power assays.

#### 2.6.1. DPPH Free Radical Scavenging Assay

DPPH° scavenging activity of *D. lycioides* organic and aqueous extracts was calculated by recording the potential of the plant extract to reduce DPPH° into DPPH-H. Briefly, 0.1 mL of plant extract (31.25–500 μg/mL) was mixed with 0.1 mM DPPH° solution in methanol. The mixture was incubated at room temperature for 1 h under aluminum foil wrapping. The absorbance was measured at 517 nm. The free radical scavenging activity of plant extract was calculated by using the following formula:

(1) $$\begin{array}{c} \text{The results DPPH scavenging activity}\left(\%\right)=\frac{\left(A_{0}-A_{1}\right)}{A_{0}}\times 100, \end{array}$$

where *A*_0_ is the absorbance of the control and *A*_1_ is the absorbance of the sample [17].

#### 2.6.2. Reducing Power Assay: Ferric Reducing Antioxidant Power

An assay was used to measure the antioxidant properties of *D. lycioides* extracts. For the reduction of ferric 2,4,6-tris(2-pyridyl)-1,3,5-triazine [Fe(III)TPTZ] to the ferrous complex, the procedure from Benzie and Strain was followed. A solution of ferric reducing antioxidant power was prepared by mixing acetate buffer (0.3 M) pH 3.6, FeCl_3_·6H _2_O (0.02 M), and TPTZ (0.01 M) in HCl (0.04 M). Under aluminum foil, 25 mL of acetate buffer, 2.5 mL of TPTZ solution, and 2.5 mL of ferric chloride hexahydrate solution were mixed and incubated for 30 min at 37°C. Briefly, 0.5 mL of (WEEAW) 1 mg/mL was mixed with 2 mL of FRAP suspension, followed by incubation at 37°C for 30 min in the dark, and the absorbance was read at 595 nm, and the following equation determined % reduction [16]:

(2) $$\begin{array}{c} \text{FRAP}\%\text{ reduction}=\left(\frac{\left(\text{AC}–\text{AS}\right)}{\text{AC}}\right)\times 100. \end{array}$$

### 2.7. Antibacterial Testing

#### 2.7.1. Microbial Culture

The antibacterial activity of the *D. lycioides* extracts was determined using the well agar diffusion method against *Enterococcus faecalis* strain (ATCC 29212), following the Clinical and Laboratory Standards Institute (CLSI) guidelines [18]. *E. faecalis* strain was cultured on brain heart agar and later inoculated and cultured in Mueller–Hinton broth.

#### 2.7.2. *E. faecalis* Susceptibility to *D. lycioides*

Antibacterial assay of *D. lycioides* organic and aqueous extracts was performed by agar well diffusion method in Mueller–Hinton agar plates. The test organisms were inoculated in Mueller–Hinton broth and incubated overnight at 37°C to adjust the turbidity to 0.5 McFarland standards giving a final inoculum of 1.5 × 10^6^ CFU/mL. Mueller–Hinton agar plates were lawn-cultured with standardized microbial culture broth. *D. lycioides* extracts of 50 μg/mL concentration were prepared in 2.5% dimethyl sulfoxide (DMSO). Three wells with a 6 mm diameter were bored in the inoculated media with the help of a 1000-μL pipette base. Each well was filled with 100 μL of *D. lycioides* extract, positive control gentamicin 50 μg/mL for bacteria, and negative control DMSO, respectively. The different treatments on the plates were allowed to diffuse into the media for about 30 min at room temperature and incubated for 24 h at 37°C. After incubation, plates were observed for the formation of a clear zone around the well which was interpreted as the antibacterial activity of tested extract. The zones of inhibition were observed and measured in mm [19]. Each experiment was repeated 3 times to generate the average inhibition per extract.

#### 2.7.3. Determination of Minimum Inhibitory Concentration (MIC) and Minimum Bactericidal Concentration (MBC) of the Plant Extracts by Resazurin Dye Method

##### 2.7.3.1. Preparation of Resazurin Solution

The resazurin solution was prepared at 0.02% (wt/vol) in deionized water according to Shatri [9]. The mixture was vortexed for 10 min. The mixture was filtered using a 0.2-μm Millipore membrane filter. The resazurin filtrate was stored at 3°C for more than 2 weeks wrapped in aluminum foil.

#### 2.7.4. Evaluation of MIC and MBC

The MIC and MBC of *D. lycioides* organic and aqueous extracts were done using the method described by Loo et al. [20] with modifications. The MIC test was done in flat-bottom 96-well microtiter plates by standard broth microdilution methods. This was done using bacterial inoculums with a concentration of 10^6^ CFU/mL. For the MIC test, 100 μL of *D. lycioides* solutions (2000-1.9 μg/mL in row 3) were added to respective wells on the 98-well plates. Row 1 contained Mueller–Hinton broth which was used as a negative control. Row 2 contained gentamicin and bacteria culture and was used as positive control. Exactly 10 μL of the bacterial inoculums in Mueller–Hinton broth were added to each well. Exactly 20 μL of the resazurin solution was added to each well and incubated at 37°C for 24 h. The plates were observed for color change. The blue or purple color was interpreted as no bacterial growth, while pink or colorless was interpreted as bacterial growth. The MIC value was taken at the lowest concentration of antibacterial agents that inhibit the growth of bacteria. The experiment was repeated 3 times. The MBC was defined as the lowest concentration of the antibacterial compound that completely killed the bacteria. The MBC test was done by culturing the MIC and the concentration that comes before and after the MIC on the Mueller–Hinton agar plates. The plates were incubated at 37°C for 24 h before observing them for any colony formation. The lowest concentration with no visible growth on the MHA plate was taken as MBC value.

#### 2.7.5. Time–Kill Curve

Time–kill assay was performed in Mueller–Hinton broth as described by Loo et al. [20]. *E. faecalis* inoculums with a concentration of 10^6^ CFU/mL were used. The *D. lycioides* with 500 μg/mL was diluted with MH broth with *E. faecalis* inoculum to obtain the final concentration of 0 × MIC, 0.5 × MIC, 1 × MIC, 2 × MIC, 4 × MIC, and 8 × MIC in the total final volume of 1 mL. The cultures were then incubated at 37°C with manual shaking every 30 min. Exactly 100 μL of *E. faecalis* cultures from each concentration were cultured on MH agar plates at times between 0 and 4 h. The experiment was done in triplicate. The number of colonies on the MH agar plates was quantified in CFU/mL after incubation at 37°C for 24 h. Gentamicin was used as a positive control, while 2.5% DMSO was used as a negative control.

### 2.8. Effect of *D. lycioides* on *E. faecalis* Biofilm

*E. faecalis* was cultured in tryptone soy broth (TSB) with 1% glucose at 37 °C for 24 h. The assay was done in flat-bottom 96-well plates. The first row in the plate containing only TSB served as control, and the next three wells had the culture media with *E. faecalis* without adding the organic or aqueous extracts of *D. lycioides* (combination extractions containing roots, twigs, and leaves). Exactly 100 μL of culture media with *E. faecalis* adjusted to 0.5 McFarland was added to the rest of the wells. All plates were incubated at 37 °C for 24 h to allow biofilm formation. The plate was rinsed with 200 μL saline, and then 100 μL of *D. lycioides* 3.9–2000 μg/mL concentrations were added to each well. All plates were incubated at 37 °C for 24 hours. The content of each well was removed and washed twice with sterile 200 μL saline; after that, the plates were allowed to dry. Biofilms were stained with 100 μL of 0.1% crystal violet, and the plates were incubated for 15 minutes. The incubated plates were then washed with 200 μL saline to remove excess dye in the wells. Exactly 200 μL of 0.5% (v/v) ethanol was added to each well to remove the dye taken up by the biofilm, and the absorbance was measured at 600 nm using a UV–vis microplate reader. The biofilm inhibition was calculated, using the equation: (%) reduction of biofilm = (total biofilm control-treated biofilm)/total biofilm control)∗100.

For treatments, gentamicin was used as a positive control. Wells without *D. lycioides* served as negative controls, and TSB medium alone served as a blank [21]. Biofilm inhibition was rated between 0% and 100%. Values below 0% were categorized as biofilm growth enhancement; between 0%–50% indicated weak antibiofilm activity, and above 50% represented good biofilm inhibition [22]. The experiment was repeated 3 times.

### 2.9. Statistical Analysis

All data were recorded and analyzed in Microsoft Excel. All experiments were done in triplicate, and experiments were repeated 3 times. Comparisons between control and experimental groups were made.

## 3. Results

### 3.1. Yield of Aqueous and Organic Extracts of *D. lycioides*

**Figure 1:**
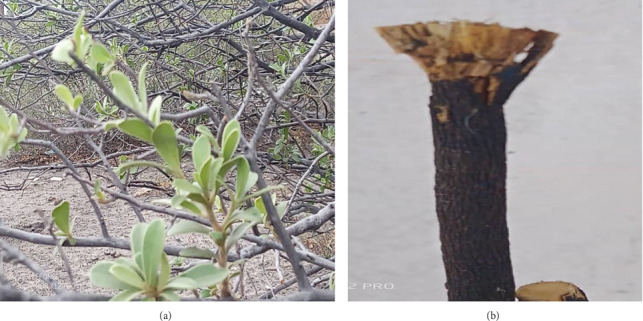
(a) *D. lycioides* plant in its natural habitat. (b) *D. lycioides* root-based chewing stick.

**Figure 2:**
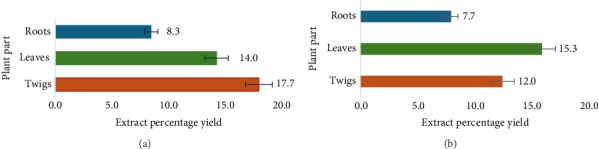
(a) Percentage yield of organic extracts of *D. lycioide*s; (b): Percentage yield of aqueous extracts of *D. lycioides*.

*D. lycioides* is found in many parts of Namibia where it grows in abundance despite its popularity in the ethnomedicinal setting.  Figure 1 shows *D. lycioides* in its natural habitat (A) and a chewing stick for brushing the teeth (B). The northern part of Namibia has a subtropical dry climate of about 22°C. The extraction of crude phytomedicine from *D. lycioides* twigs in organic solvents and leaves in aqueous solvents showed yields above 15%. Methanol extracts of twigs (17.7%) (Figures 2(a), 2(b)) were the highest, while aqueous leaf extract had a yield of 15% (Figures 2(a), 2(b)).

### 3.2. Total Phenolic Contents

**Figure 3:**
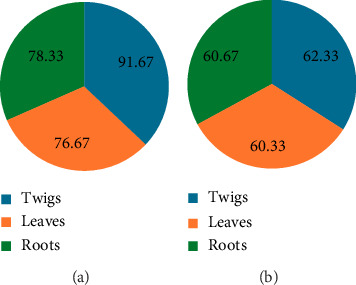
Total phenolic content of (a): organic and (b): aqueous extracts of *D. lycioides* in mg (GAE)/g.

The methanol extract carries higher quantities of total phenol and total flavonoids than the aqueous extracts. The highest total phenol content recorded for organic extracts (91.67 ± 0.578 mg (GAE)/g) and the highest for aqueous extracts (62.33 ± 0.57 mg (GAE)/g) were both from twig extracts (Figures 3(a) and 3(b)). This could explain why twigs and roots are preferred to using twigs for preparing toothbrush sticks in the traditional setting.

### 3.3. Total Flavonoid Contents

**Figure 4:**
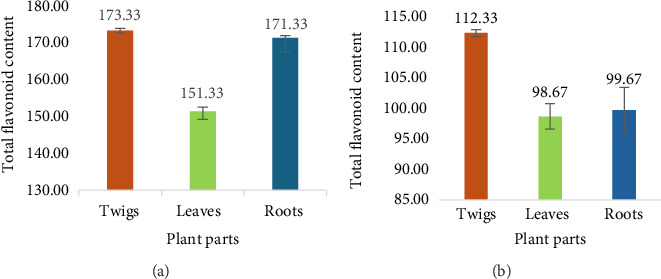
Total flavonoids content of (a): organic and (b): aqueous extracts of *D. lycioides* in mg (QE)/g.

The highest total flavonoid content of 173.33 ± 0.56 mg (QE)/g was reported in methanol twig extracts of *D. lycioides*, while aqueous leaves extract showed the lowest total flavonoid content of 98.67 ± 2.08 mg (QE)/g as indicated in Figures 4(a) and 4(b), respectively.

### 3.4. Total Antioxidant Testing

**Figure 5:**
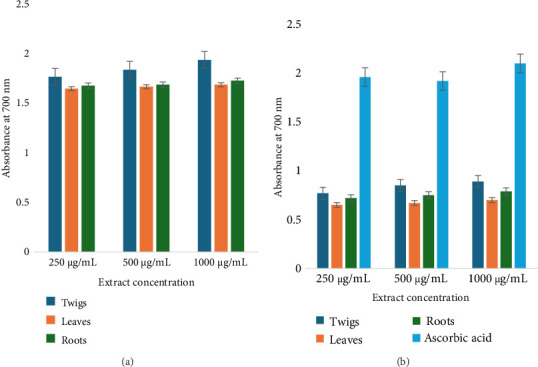
Antioxidant activity of (a): organic and (b): aqueous extracts of *D. lycioides* and positive control ascorbic acid.

**Figure 6:**
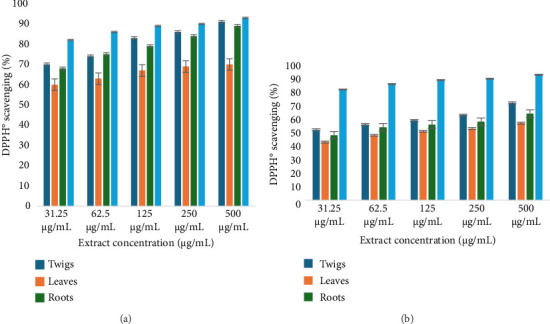
DPPH scavenging activity of (a): organic and (b): aqueous extracts of *D. lycioides* and positive control ascorbic acid.

Antioxidant analysis showed a concentration-dependent effect in both organic and aqueous extracts. Among organic extracts, twig extracts showed higher absorbance between 1.77 ± 0.085 and 1.84 ± 0.085 nm as shown in Figures 5(a) and 5(b). The reducing potential of the standard antioxidant, ascorbic acid, showed a comparative absorbance value, especially with twig extracts (1.96–2.1 ± 0.095) at similar concentration levels. All the assessed organic and aqueous extracts of *D. lycioides* revealed a gradual fading in purple-colored radical DPPH° into the yellow-colored DPPH-H by increasing the extract concentration. Methanol twig extracts showed the highest scavenging ability (91.0 ± 0.71%). There is a statistical difference between the aqueous extracts and positive control ascorbic acid (*p* value < 0.05); however, there was no statistically significant difference between the antioxidant activity of organic extracts and positive control ascorbic acid (*p* value > 0.05). All aqueous extracts showed lower scavenging ability than organic extracts (Figures 6(a) and 6(b)). Ascorbic acid was used as the standard in the antioxidant test analysis due to its well-established antioxidant properties. It showed comparable antioxidant activity in both aqueous and organic environments under the experimental conditions. Preliminary tests conducted in this study confirmed that differences in activity existed between the two solvent systems. Therefore, to streamline data presentation, a single representative result for ascorbic acid's antioxidant activity in both environments is presented in Figure 5(b).

### 3.5. Antibacterial Activity

**Figure 7:**
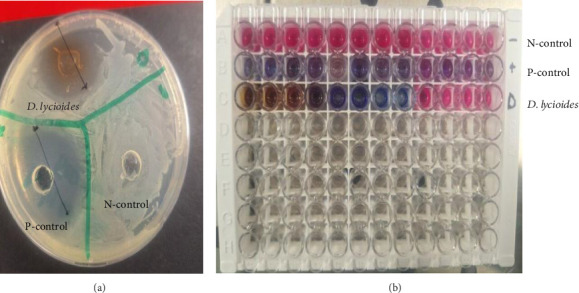
(a): Visible clear zone produced by *D. lycioides* organic extract. (b): MIC of *D. lycioides*, N-control (2.5% DMSO), and P-control (gentamicin).

The antibacterial activity of organic and aqueous extracts of *D. lycioides* was determined against the root canal-infecting *E. faecalis.* The results for the agar well diffusion test, of *D. lycioides* aqueous and organic extracts, as well as the controls, are summarized in Table 1. For the good diffusion test, the presence of a clear zone around the well containing *D. lycioides* suggested that *D. lycioides* possessed antibacterial activity due to its ability to inhibit the growth of *E. faecalis*. The visible clear zone produced by organic extracts of *D. lycioides* and controls against *E. faecalis* is shown in Figure 7(a), while the MIC results are shown in Figure 6(b). The inhibition zone of *D. lycioides* organic extract shows an average zone of inhibition of 28 ± 0.82 mm. Organic extracts of *D. lycioides* showed more potent antibacterial activity than aqueous extracts with MICs of 15.6 ± 0.00 and 250 ± 0.00 μg/mL and MBCs of 31.8 ± 0.00 and 500 ± 0.00 μg/mL, respectively (Table 1).

Resazurin dye was used in the study as an indicator in the determination of cell growth. As observed in Figure 7(b), oxidoreductases within viable cells reduced the resazurin salt to resorufin and changed the color from blue nonfluorescent to pink (negative control) and remained blue/violet (gentamicin and *D. lycioides*).

### 3.6. Kill–Time Curve

**Figure 8:**
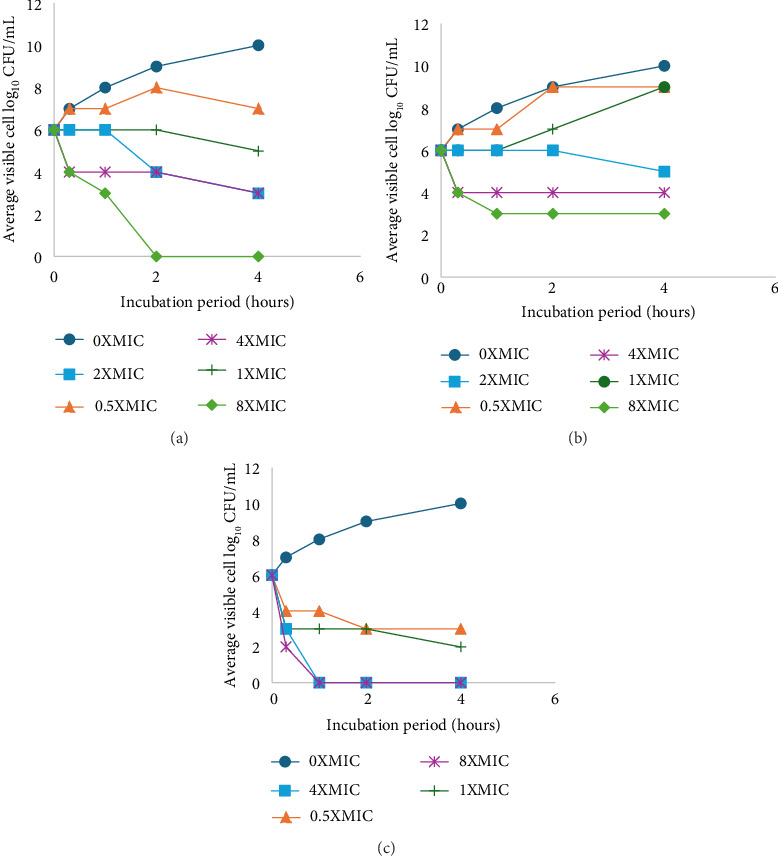
Time–kill plots of (a) *D. lycioides* organic extract, (b) *D. lycioides* aqueous extract, and (c) gentamicin against *E. faecalis* at different concentrations and time length.

**Figure 9:**
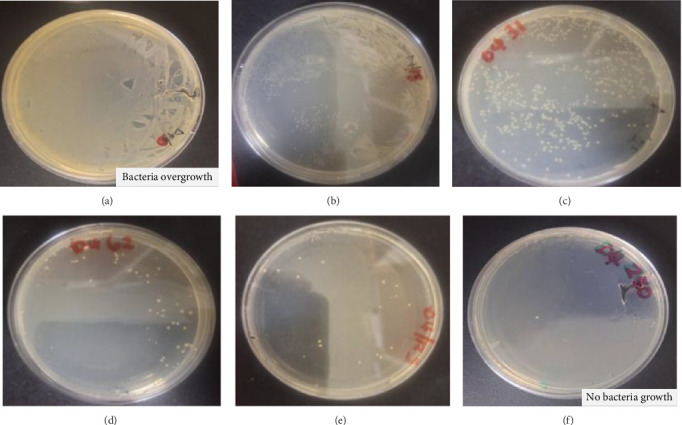
The time–kill kinetics profile of different concentrations of *D. lycioides* organic extract 8(a-f: 0 × MIC–8 × MIC) after 4 h of incubation with *E. faecalis*.

The time–kill activity of *E. faecalis* by *D. lycioides* organic and aqueous extracts (combination extractions containing roots, twigs, and leaves). and positive control gentamicin is shown in Figures 8(a), 8(b), 8(c). The organic extracts of *D. lycioides* showed bactericidal properties. The bactericidal endpoint of *D. lycioides* organic extracts for *E. faecalis* was reached after 4 h of incubation at 8 × MIC (124.8 μg/mL) as depicted in Figure 9. The bactericidal endpoint of gentamicin was reached after 2 h at 4 × MIC (31.2 μg/mL). However, the aqueous extract of *D. lycioides* showed bacteriostatic properties as they could not reach an endpoint during the time investigated as shown in Figure 8. There was a significant difference between the kill time of *D. lycioides* and gentamicin (*p* ≤ 0.05).

### 3.7. Effect of *D. lycioides* on *E. faecalis* Biofilm Formation

**Figure 10:**
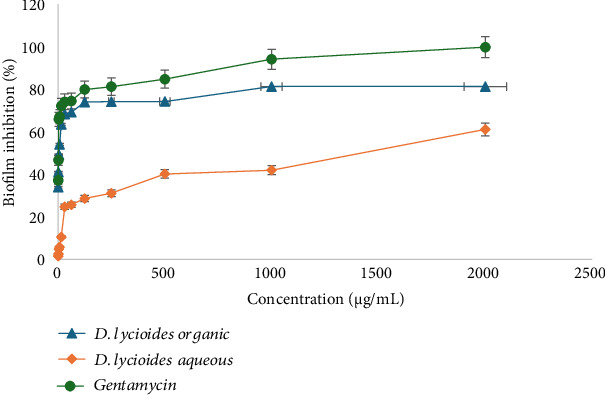
Percentage of biofilm inhibition by blue: organic extract, orange: aqueous extract, green: gentamycin.

All the extracts had various levels of biofilm inhibitory activity against *E. faecalis*. The organic extracts (combination extractions containing roots, twigs, and leaves) showed antibiofilm properties between 49.3 and 81.27± against *E. faecalis* at concentrations ranging between 3.7 and 2000 μg/mL indicating strong biofilm inhibition. However, aqueous extracts only showed more than 50% biofilm inhibition at 2000 μg/mL. This demonstrates that aqueous extracts have weak biofilm inhibition properties. The biofilm inhibition of organic extracts of *D. lycioides* was comparable to gentamicin and was used as a positive control as indicated in (Figure 10).

## 4. Discussion

### 4.1. Total Quantification of Phytoconstituent Compounds and Antioxidant Testing

Methanol and water are reported to have higher lipophilic behavior than other solvents. This is associated with a strong potential to extract phytochemicals with biological effects. Moreover, good yield in aqueous leaf extracts might be due to the presence of the phytochemicals that bear electronegative functional groups making the compound hydrophilic [11]. The yield of a plant extract allows for calculating the quantity needed for biological assays to compare plants for bioprospecting and scale-up in cases of future product development [23].

These findings on total quantifications agree with the findings of Ha et al. [24]. Moreover, other subspecies of *lycioides* such as *Rhamnus lycioides* are reported to contain high phenolic and flavonoids contents [25]. Literature showed that multiple hydroxyl functional groups present in phenolics, and flavonoids are responsible for their biological and antioxidant activities; hence, these compounds could be responsible for the effectiveness of the decoctions prepared in the traditional setting in alleviating tooth pain and treating oral infections [26].

The results show that *D. lycioides* extracts are rich in phenolic and flavonoid compounds. The phytomedicine prepared has shown potent antioxidant activity with scavenging abilities of over 45% in both aqueous and organic phytomedicine prepared. *D. lycioides* extracts also showed potent antibacterial activity against *E. faecalis* with a MIC of 15.6 μg/mL. The extract could also inhibit biofilm formation. This makes it a promising natural source of compounds for eliminating E. *faecalis.* The limitation of this study is that it made use of a reference strain and not a clinical strain of E. faecalis. The extracts should be used for further analysis to evaluate the possibilities of enhancing efficacy within appropriate in vitro and in vivo models. There is also a need to isolate and purify the compounds from the extracts responsible for the antimicrobial properties which may serve as potential antibiotics.

It is interesting to notice that extracts of *D. lycioides* showed higher total phenolic and flavonoid contents also showed higher antioxidant properties, as this could influence the biological activities of the extracts. These findings are consistent with the findings of Abbas et al. [11]. Previous studies described that the potent free radical scavenging activity of plant extracts correlates with their TPC [27], and these results agree with their findings in this study. This is the first report on the kill time of *D. lycioides.* Natural antioxidants such as polyphenols and carotenoids exhibit a wide range of biological effects, including anti-inflammatory, anti-aging, anti-atherosclerosis, and anticancer [28, 29]. Inflammation is one symptom that indicates oral tissue infection by pathogenic organisms, and it presents with unbearable pain. Hence, antioxidant properties present in the chewing material could offer anti-inflammatory properties which are crucial for proper healing.

### 4.2. Bacteriostatic and Bactericidal Activity of *D. lycioides*

According to Gado [30], MIC values less than 0.1 mg/mL are considered as good antimicrobial activity; MIC of 0.1 to 0.5 mg/mL is moderate antimicrobial activity, while MIC of 0.5 to 1 mg/mL is weak antimicrobial activity and MIC of greater than 1 mg/mL is considered inactive. Therefore, the findings of this study indicate promising antibacterial activity of *D. lycioides* with potent MICs ≤ 0.25 mg/mL. Organic extracts of *D. lycioides* showed more potent antibacterial activity than aqueous extracts. This contradicts the ethnomedicinal preparation techniques of herbal remedies that are normally done using water. This supports the findings of Kanyemba et al. [12], regarding the promising antibacterial properties of D. *lycioides* against *Mycobacterium avium* and *Staphylococcus aureus*. The findings of this study also agree with those of Kanyemba et al. [12] regarding the broad-spectrum potent antibacterial properties of *D. lycioides.* Bagla et al. [29] also reported that *D. lycioides* have antibacterial activity against *Staphylococcus aureus, Streptococcus faecalis,* and *Escherichia coli.* This makes it a good source of antibacterial treatments for different pathogens. In another study by Cai et al. [31], compounds such as naphthalene glycosides and diospyroides have been identified in *D. lycioides* and linked to their efficacy against oral cariogenic bacteria, namely *Streptococcus mutans* and *Streptococcus sanguinis*, as well as periodontal pathogens, namely *Porphyromonas gingivalis* and *Prevotella intermedia* at MICs ranging from 0.019 to 1.25 mg/mL According to Hochman et al. [30], organic extracts have more phytomedicine which is the attributing factor to the ethnomedicinal uses and potent antibacterial activity observed in *D. lycioides* organic extract against *E. faecalis*. The extracts prepared in this study could be potential sources of compounds for treating *E. faecalis-induced* periodontitis, peri-implantitis, and caries.

Organic solvents like ethanol, methanol, or acetone are better at dissolving and extracting nonpolar or moderately polar phytochemicals such as flavonoids, terpenoids, and naphthoquinones, which are often responsible for strong antimicrobial activity. In contrast, water tends to extract mainly polar compounds like sugars, tannins, and certain phenolics, which may have weaker or less targeted antibacterial effects. Despite the aqueous extract being used at much higher concentrations (up to 2000 μg/mL), it still failed to achieve complete (100%) biofilm inhibition. This suggests that the bioactive compounds essential for disrupting E. faecalis biofilms are either poorly water-soluble or present in very low concentrations in the aqueous extract. Meanwhile, the organic extract, even at much lower concentrations, achieves bactericidal levels (MBC of 31.8 μg/mL), demonstrating its superior potency.

There are limited data on the time–kill kinetic studies of medicinal plants despite their significance in filling the knowledge gap in the literature regarding the effective dosage when using medicinal plants. Several reports of other natural products such as mushrooms and actinomycetes extracts have been reported [32, 33]. From that time, the killing analysis exposed the degree of time-dependent differences between aqueous and organic extracts. While aqueous extracts showed the organic extracts of *D. lycioides* showed bactericidal properties with a kill time of 4 h, aqueous extracts showed bactericidal properties since a kill–time point could not be reached. Surachai et al. [34] have shown that plant secondary metabolites differences in aqueous and organic extracts determine the effect of the extract on microbial infection. With increasing cases of *E. faecalis* antibiotic resistance patterns [35], these extracts can be potential sources of future antimicrobials for treating *E. faecalis*-induced periodontal infections.

### 4.3. Biofilm Eradication Properties of *D. lycioides*

The frequency of *E. faecalis* in primary endodontic infections is 4%, while persistent periapical lesions are 77%. This is because *E. faecalis* can survive as a single microorganism and form a major biofilm, which causes endodontic treatment failures [36]*. D. lycioides* extracts prepared in this study have shown potent biofilm-inhibiting properties. Biofilm inhibition properties of *D. lycioides* against *E. faecalis* suggest that adding phytomedicine before biofilm formation eliminates planktonic cells and may reduce the polystyrene surface adherence, which becomes less susceptible to cell adhesion [37, 38]. Furthermore, the modification of *E. faecalis* surface proteins caused by their interactions with medicinal plants is well documented in plants such as *Origanum majorana, Rosmarinus officinalis*, and *Thymus zygis* and they agreed with our findings for *D. lycioides* extracts. Inhibition is due to the adhesion of the bacterium to the polystyrene surface, which is the initial attachment phase. These findings agree with that of Ben et al. [33] regarding the biofilm inhibition properties of plant-based products. Furthermore, according to Mouafo et al. [39, 40], the inhibition of bacterial signaling systems and the downregulation of the expression of genes that are involved in biofilm formation occur due to the degradation of the membrane potential of bacterial cells embedded in the biofilm. This could be the mechanism used by *D. lycioides* in eradicating biofilm observed in this study. Furthermore, its ability to inhibit biofilm formation, even partially, suggests that with formulation optimization or adjunctive use, the aqueous extract of *D. lycioides* may serve as a natural, plant-derived alternative or complement to conventional irrigants like chlorhexidine or sodium hypochlorite.

This study highlights the antibacterial and antibiofilm potential of *D. lycioides* extracts against *E. faecalis*, a persistent pathogen commonly associated with root canal failures. The organic extract exhibited significantly higher antibacterial activity, reflected in its low MIC and MBC and kill kinetic values, likely due to the efficient extraction of nonpolar phytochemicals with potent bioactivity. However, its application in clinical settings, particularly as a root canal irrigant, may be limited by the cytotoxicity or incompatibility of organic solvents. Hence, further studies should be conducted to evaluate the toxicity profiles of both aqueous and organic extracts. Conversely, the aqueous extract, despite requiring higher concentrations and showing lower efficacy, offers advantages in terms of biocompatibility and safety, making it a more suitable candidate for intracanal application. The documented biofilm inhibition properties of *D. lycioides* suggest its potential as a natural irrigant, especially if optimized or used in combination with other agents such as nanomaterial to enhance its uptake mucoadhesive properties, bioavailability, and reduce dosage. Therefore, *D. lycioides* in both aqueous and organic extracted form hold promise as a plant-based adjunct or alternative in endodontic disinfection protocols. Therefore, further investigation into formulation enhancements, delivery methods, and synergistic effects with existing treatments should be evaluated to maximize the potential applications of *D. lycioides* in endodontic treatments.

## 5. Conclusion

The results show that *D. lycioides* extracts are rich in phenolic and flavonoid compounds. The phytomedicine prepared has shown potent antioxidant activity with scavenging abilities of over 45% in both aqueous and organic phytomedicine prepared. *D. lycioides* extracts also showed potent antibacterial activity against *E. faecalis* with a MIC of 15.6 μg/mL. The extract could also inhibit biofilm formation. This makes it a promising natural source of compounds for eliminating E. *faecalis.* The extracts should be used for further analysis to evaluate the possibilities of enhancing efficacy within appropriate in vitro and in vivo models. There is also a need to isolate and purify the compounds from the extracts responsible for the antimicrobial properties which may serve as potential antibiotics.

## Figures and Tables

**Table 1:** The diameter of zone inhibition, MIC, and MBC values of *D. lycioides* and controls.

Treatment	Average diameter of inhibition zone (mm)	MIC (μg/mL)	MBC (μg/mL)
*D. lycioides* organic	28 ± 0.82	15.6 ± 0.00	31.8 ± 0.00
*D. lycioides* aqueous	13 ± 0.82	250 ± 0.00	500 ± 0.00
Gentamicin	32 ± 1.63	3.9 ± 0.00	3.9 ± 0.00
2.5% DMSO	0 ± 0.00	0 ± 0.00	0 ± 0.00

## Data Availability

Data will be available upon request from the corresponding author.
